# High efficacy of CpG-ODN, Cetuximab and Cisplatin combination for very advanced ovarian xenograft tumors

**DOI:** 10.1186/1479-5876-11-25

**Published:** 2013-01-29

**Authors:** Michele Sommariva, Michelandrea de Cesare, Alessandra Meini, Alessandra Cataldo, Nadia Zaffaroni, Elda Tagliabue, Andrea Balsari

**Affiliations:** 1 Dipartimento di Scienze Biomediche per la Salute, Università degli Studi di Milano, via Mangiagalli 31, 20133, Milan, Italy; 2 Molecular Pharmacology Unit, Fondazione IRCCS - Istituto Nazionale Tumori, via Amadeo 42, 20133, Milan, Italy; 3 Molecular Targeting Unit, Fondazione IRCCS - Istituto Nazionale Tumori, via Amadeo 42, 20133, Milan, Italy

**Keywords:** CpG-ODN, TLR9, Ovarian cancer, Ascites, Monoclonal Antibody, Cisplatin

## Abstract

**Background:**

To mimic clinical treatment situations in advanced human ovarian disease, we tested the efficacy of CpG-oligodeoxynucleotides (CpG-ODN), synthetic DNA sequences recognized by Toll-like receptor 9 and able to induce innate/adaptive immune responses, in combination with other possible therapeutic reagents in ovarian carcinoma ascites-bearing athymic mice.

**Methods:**

Mice injected i.p. with IGROV-1 ovarian cancer cells were treated at different stages of ascites progression for 4 weeks with CpG-ODN, alone or in combination with Bevacizumab, Polyinosinic:Polycytidylic acid (Poly(I):Poly(C)), Gefitinib, Cetuximab and Cisplatin. Median survival time (MST) was calculated for each group. IGROV-1 cells treated or not with Cetuximab were assayed for antibody-dependent cellular cytotoxicity by ^51^Cr-release assay, and for macrophage antibody-dependent cell-mediated phagocytosis by flow cytometry.

**Results:**

In mice treated when ascitic fluid began to accumulate, CpG-ODN combined with Bevacizumab, Poly(I):Poly(C) or Gefitinib did not significantly increase MST as compared with that using CpG-ODN alone, whereas MST in mice treated with CpG-ODN plus Cetuximab was significantly increased (>103 days for combination vs 62 days for CpG alone; P = 0.0008), with 4/8 mice alive at the end of the experiment. In experiments in mice showing increased abdominal volume and body weight (27.9 ± 0.8 g after vs 23 ± 1.1 g before tumor cell injection), treatment with Cisplatin in addition to CpG-ODN/Cetuximab led to significantly increased MST (105.5 days; P = 0.001), with all mice still alive at 85 days, over that using CpG-ODN/Cetuximab (66 days), Cetuximab/Cisplatin (18.5 days), Cisplatin (23 days) or saline (16 days). At a very advanced stage of disease (body weight: 31.4 ± 0.9 g), when more than half of control mice had to be sacrificed 6 days after starting treatments, the triple-combination therapy still increased MST (45 days; P = 0.0089) vs controls.

**Conclusions:**

CpG-ODN combination therapies that enhance the immune response in the tumor microenvironment and concomitantly target tumor cells are highly efficacious even in experimental advanced malignancies. Although differences in the distribution of TLR9 in mice and humans and the enrichment of this receptor on innate immune cells of athymic mice must be considered, our results indicate a promising strategy to treat ovarian cancer patients with bulky ascites.

## Introduction

Advanced tumor disease in humans is generally much less responsive than limited disease to most anti-cancer therapies. Testing of new cancer therapies in preclinical models for advanced human ovarian cancer remains rare. Treatment usually involves minimal disease and therapy-naïve tumors, possibly accounting for the failure to reproduce encouraging preclinical results in subsequent Phase I/Phase II clinical trials [1].

CpG-ODNs are synthetic DNA sequences that mimic bacterial DNA and are recognized by the Toll-like receptor 9 (TLR9) member of the TLR family as a “danger signal” [2,3] consisting of pathogen-associated molecular patterns or stress-induced self molecules [4]. Interaction of CpG-ODN with TLR9 can result in the generation of both innate and adoptive immune responses, either of which have the potential to significantly impact tumor growth. CpG-ODN have demonstrated antitumor activity in different animal models [5,6] and in patients with malignant melanoma, renal carcinoma and recurrent or refractory lymphoma [7-10]. However, although both preclinical and early clinical trials suggest the value of CpG-ODN as a component of various approaches to cancer therapy, clinical development of this recently discovered novel class of immunostimulatory agents is in the incipient stage and much remains unknown about their optimal use [11]. Our recent studies revealed that local, but not systemic, and daily, but not weekly, stimulation of immune effector cells by CpG-ODN targeted immunotherapy inhibited ascites production and significantly prolonged survival in human ovarian carcinoma ascites-bearing athymic mice [12]. However, CpG-ODN monotherapy might be sufficient to induce tumor regression only in small tumors; for larger tumors, CpG-ODN needs to be combined with other effective anti-tumor strategies [8]. Indeed, further evaluation in our advanced-stage human ovarian tumor-bearing mouse model showed that CpG-ODN combined with the DNA-damaging chemotherapeutic drug cisplatin, which is currently used in ovarian cancer patients [13], significantly increased the lifespan of mice compared with the individual treatments [14]. Interestingly, we found that CpG-ODN treatment at the tumor site down-modulated DNA repair genes in tumor cells [14]. The findings that peri-tumoral delivery of CpG-ODN is critical in DNA repair gene down-modulation in tumors but that CpG-ODN does not interact directly with the tumor cells to induce this down-modulation points to the importance of the activation of TLR9-positive cells. Because CpG-ODN-induced activation of TLR9-positive cells in the tumor microenvironment might also induce increased therapeutic activity of other drugs with different mechanisms of action, we used IGROV-1 ovarian carcinoma ascites-bearing athymic mice to evaluate the efficacy of CpG-ODN combined with: the monoclonal antibody (MAb) Bevacizumab targeting the vascular endothelial growth factor (VEGF) [15,16], which is reportedly overexpressed in ovarian cancer [17-19] and which regulates angiogenesis as an important component of ovarian cancer growth [16,20]; Poly(I):Poly(C), a TLR3 agonist reported to synergize with TLR9 ligand to mediate enhanced activation of innate immunity [21]; MAb Cetuximab, which targets the ligand-binding domain of the epidermal growth factor receptor (EGFR) frequently expressed in ovarian cancer cells [20]; and Gefitinib, a tyrosine kinase inhibitor of EGFR. Our observation that CpG-ODN plus Cetuximab strongly increases survival over that seen with either reagent alone led to analysis of the efficacy of a therapeutic protocol involving CpG-ODN, Cetuximab and Cisplatin in mice with very advanced ovarian tumors.

## Materials and methods

### Mice

Eight- to 12-week-old female Swiss nude (athymic) mice (Charles River, Calco, Italy) were maintained in laminar-flow rooms at constant temperature and humidity, with food and water given *ad libitum*. Experiments were approved by the Ethics Committee for Animal Experimentation of the Fondazione IRCCS Istituto Nazionale Tumori of Milan according to institutional guidelines.

### ODNs and drugs

Purified, phosphorothioated ODN1826 (5’-TCCATGACGTTCCTGACGTT-3’) containing CpG motifs was synthesized by TriLink Biotechnologies (San Diego, CA, USA). Phosphorothioate modification was used to reduce susceptibility of the ODN to DNase digestion, thereby significantly prolonging its *in vivo* half-life. The following drugs were used: Bevacizumab (Roche, Basel, Switzerland); Poly(I)Poly(C) (Amersham Biosciences, Piscataway, NJ, USA); Cetuximab (Erbitux®, Merck Serono, Darmstadt, Germany); Gefitinib (LC Laboratories, Woburn, MA, USA); and Cisplatin (Teva Italia, Milan, Italy). Lyophilized ODN1826 and Poly(I):Poly(C) were dissolved in sterile water at a concentration of 10 mg/ml and 2 mg/ml, respectively, and stored at −20°C until use. Gefitinib was dissolved in DMSO (10% v/v final concentration) and diluted in carboxymethylcellulose (0.25% w/v) to a final concentration of 10 mg/ml. Bevacizumab, Cetuximab and Cisplatin (purchased in their commercial formulation) were diluted in 200 μl of sterile saline at the indicated concentrations just before administration.

### Cell culture

For *in vitro* experiments, human IGROV-1 ovarian tumor cells (gift from Dr. J. Benard, Institute Gustave Roussy, Villejuif, France) [22] were cultured in RPMI medium 1640 supplemented with 10% FCS (Sigma, St. Louis, MO) and 2 mM glutamine (Cambrex, East Rutherford, NJ, USA) (complete medium). Mouse leukemic monocyte/macrophage RAW 264.7 cells (American Type Culture Collection) were cultured in DMEM (Sigma) supplemented with 10% FCS (Sigma) and 2 mM glutamine (Cambrex). All cultures were maintained at 37°C in a 5% CO_2_ humidified environment.

### Therapy studies

IGROV-1 human ovarian carcinoma cells were adapted to growth i.p. and maintained by serial i.p. passages of ascitic cells into healthy mice as described [22]. Mice were injected i.p. with 2.5 × 10^6^ ascitic cells in 0.2 ml of saline and treated 7 days later, when ascitic fluid began to accumulate, with CpG-ODN i.p. daily for 4 weeks (20 μg/mouse) in combination with: Bevacizumab (5 mg/kg i.p. at 3–4 day intervals); Poly(I):Poly(C) (20 μg/mouse i.p. at 2–3 day intervals); Gefitinib (100 mg/kg *per os,* 5 days/week); or Cetuximab (1 mg/mouse i.p. at 3–4 day intervals). Single agents were also included and control mice received saline.

In other experiments, mice with evident and established ascites were selected on the basis of a similar body weight (mean 27.9 ± 0.84 g, 31.4 ± 0.9 g, first and second experiment, respectively) from large groups of mice injected i.p. 11–12 days before IGROV-1 cell injection and randomly divided into saline-treated (controls) and groups treated with CpG-ODN, Cetuximab (both with the schedules reported above) and Cisplatin (3 mg/Kg i.p., once weekly for 4 weeks) or their combinations. Experimental groups (5–12 mice/group) were inspected daily for ascites formation and weighed three times weekly. Mice were individually sacrificed by cervical dislocation prior to impending death. Day of sacrifice was considered day of death, and the median day of death (median survival time; MST) was calculated for each group. Anti-tumor activity was assessed as the ratio of MST in treated vs. control mice × 100 (T/C%).

### Flow cytometry

IGROV-1 cells were exposed to Cetuximab (5 μg/m) for 72 h or left untreated, collected and incubated for 30 min at 4°C with anti-MICA, -MICB, -ULBP1, -ULBP2, ULBP4, -CD112, -CD155, -ICAM-1, and HLA-E antibodies (R&D Systems; Minneapolis, MN. USA), followed by incubation with anti-mouse Alexa Fluor 448-conjugated reagent (Invitrogen). Samples were analyzed by gating on live cells using FACSCanto II system (Becton-Dickinson, San Jose, CA) and BD FACSDiva™ software (Becton-Dickinson). EGFR expression levels on IGROV-1 cells were determined after incubation for 30 min at 4°C with Cetuximab (10 μg/ml), followed by incubation with anti-mouse Alexa Fluor 448-conjugated antibody (Invitrogen).

### Antibody-dependent cellular cytotoxicity (ADCC) assay

IGROV-1 cells were treated or not (controls) with Cetuximab (5 μg/ml for 72 h) and labeled with 100 μCi ^51^Cr (PerkinElmer, Waltham, Massachusetts) for 1 h at 37°C. After 3 washes with PBS-5% FCS, cells were co-incubated for 4 h at 37°C with PBMC from 12 healthy donors (effector:target ratio 50:1) in 200 μl RPMI 1640 complete medium in triplicate 96-well U-bottomed plates in the presence of saturating concentrations of Cetuximab (10 μg/ml). Radioactivity of the supernatant (80 μl) was measured with a Trilux Beta Scintillation Counter (PerkinElmer). Percent specific lysis was calculated as: 100 × (experimental cpm - spontaneous cpm)/ (maximum cpm - spontaneous cpm).

### Phagocytosis assay

Macrophage antibody-dependent cell-mediated phagocytosis (ADCP) was assessed by flow cytometry [23]. Murine RAW264.7 effector cells were labeled with PKH26 (Red Fluorescent Cell Linker Mini Kit), while IGROV-1 target cells were labeled with PKH67 (Green Fluorescent Cell Linker Mini Kit) according to the manufacturer’s instructions (Sigma). IGROV-1 cells were then seeded in tissue culture flasks and exposed to Cetuximab (5 μg/ml for 72 h) or left untreated. At the end of treatment, target and effector cells were mixed at E:T ratio of 3:1 in complete medium and incubated for 12 h at 37°C in overload conditions of monoclonal antibody (10 μg/ml). Cells were collected, washed, resuspended in cold Ca^2+^- and Mg^2+^-free Dulbecco’s PBS and analyzed by flow cytometry (FACSCanto II, Becton-Dickinson). Phagocytosis of IGROV-1 cells by RAW264.7 cells was evaluated in triplicate as percentage and intensity of macrophages positive for green fluorescence in at least three separate experiments.

### Statistical analysis

Percent survivorship was estimated by the Kaplan-Meier product limit method and compared with the log-rank test.

## Results and discussion

**Figure 1:**
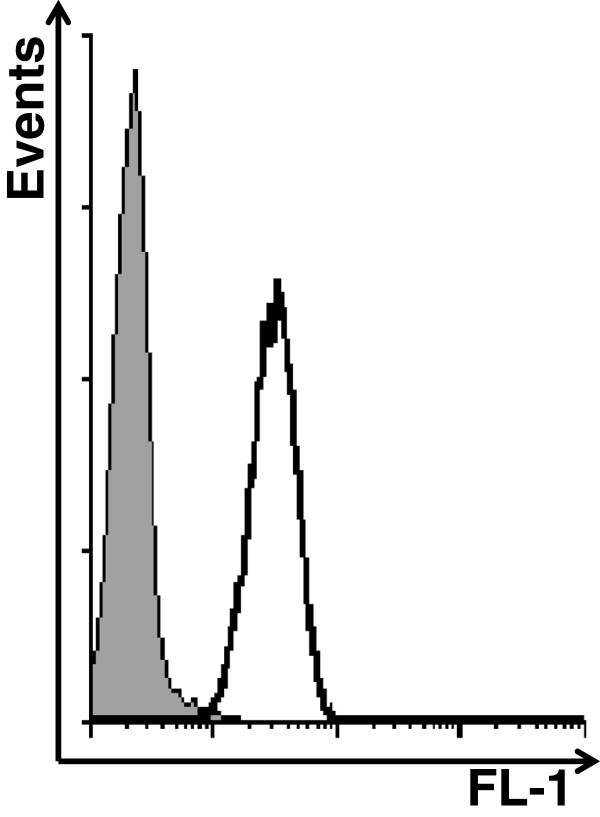
**EGFR expression in IGROV-1 cells.** Expression of EGFR in IGROV-1 cells evaluated by FACS. Thin and bold lines represent Alexa Fluor 488-secondary antibody and Cetuximab, respectively.

**Figure 2:**
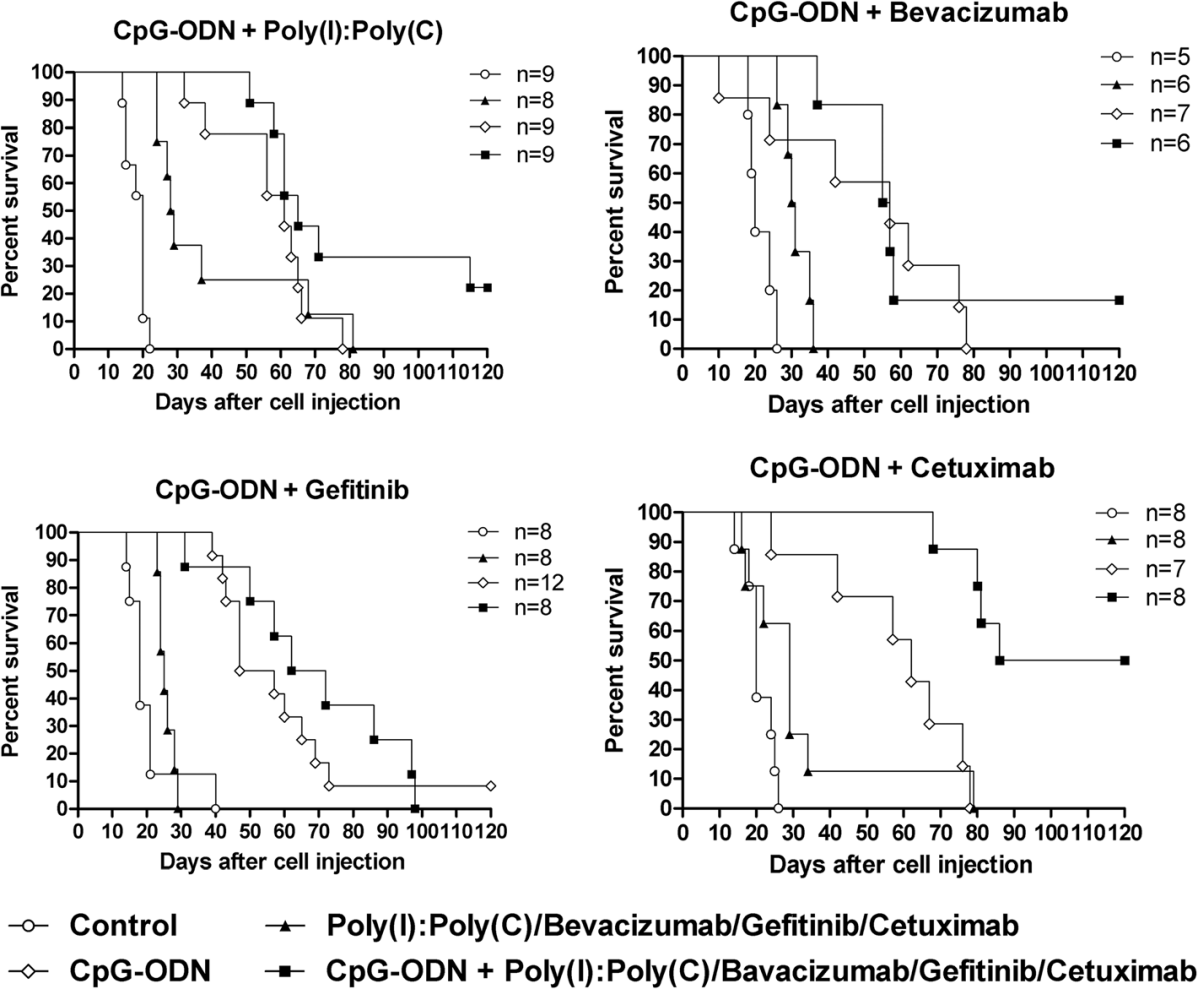
**Kaplan-Meier plot of percent survival over time among IGROV-1 ovarian tumor-bearing mice.** At 7 days after tumor cell injection, mice were treated i.p. with CpG-ODN (20 μg/mouse, 5 days/week for 4 weeks) in combination with: Poly(I):Poly(C) (20 μg/mouse at 2- to 3-day intervals); Bevacizumab (5 mg/Kg at 3- to 4-day intervals); Gefitinib (100 mg/mouse, 5 days/week) or Cetuximab (1 mg/mouse at 3- to 4-day intervals). Single agents were also tested. Control mice received saline. N = number mice/group.

The efficacy of CpG-ODN in combination with Poly(I):Poly(C), Bevacizumab, Cetuximab, or Gefitinib was evaluated in mice injected i.p. with IGROV-1 cells (which express EGFR, Figure 1) and treated on day 7, when ascitic fluid began to accumulate. Repeated i.p. CpG-ODN treatments plus Poly(I):Poly(C) induced negligible effects on MST (65 days, T/C% 325) compared with CpG-ODN treatment alone (61 days, T/C% 305), with 2 of 9 mice from the combined treatment group showing long-term survival at the end of the experiment (120 days) (Figure 2). The previously observed synergy between the two immune modulators [21] was not seen, possibly due to the schedule of CpG-ODN administration; indeed, daily CpG-ODN administration might induce massive innate cell activation hardly expandable by other immune modulators.

Repeated i.p. CpG-ODN treatments plus anti-VEGF Bevacizumab (Figure 2) also did not enhance the effect of CpG-ODN treatment alone (MST 56 days for the combination vs 62 days for CpG-ODN alone). Of note, Bevacizumab as a single agent delayed the onset of ascites (data not shown), consistent with recent preclinical and clinical data and suggesting that targeting VEGF might suspend ascites production resulting from peritoneal metastasis [24].

The combination of the EGFR tyrosine kinase inhibitor Gefitinib and repeated i.p CpG-ODN induced only a modest increase (p = 0.4099) in lifespan (MST 67 days, T/C% 372) compared with that in mice treated with CpG-ODN alone (MST 52 days, T/C% 289), whereas survival was significantly increased (p = 0.0008) in mice treated with CpG-ODN plus Cetuximab (MST > 103 days) versus those treated with CpG-ODN alone (MST 62 days) (Figure 2), with 4 of 8 mice still alive at the end of the experiment. The latter finding most likely reflects CpG-ODN-induced recruitment and activation of immune effector cells at the site of tumor growth [25-27]; nevertheless these impressive results in a model in which the antibody alone had slight effect might be also related to a Cetuximab-induced increase in susceptibility of tumor cells to CpG-ODN-activated effector cells involved in ADCC and/or in phagocytosis [28,29].

**Figure 3:**
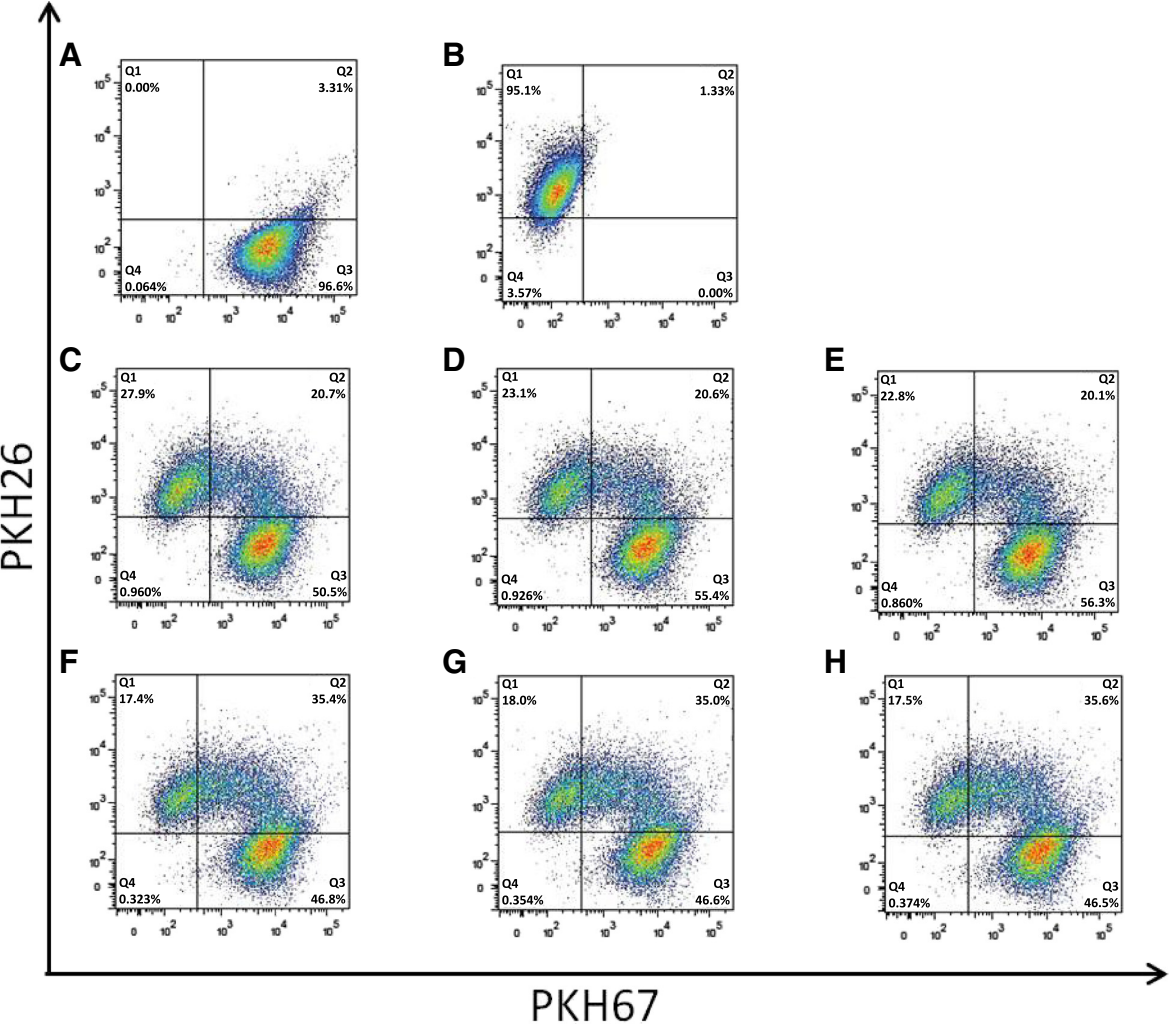
**Effect of Cetuximab pretreatment on phagocytosis of IGROV-1 cells.** IGROV-1 target cells were stained green with PKH67 (**A**, right lower quadrant) and RAW264.7 effector cells were stained red with PKH26 (**B**, left upper quadrant). Tumor targets were pre-incubated for 72 h with 5 μg/ml Cetuximab (**F**,**G**,**H**) or left untreated (**C**,**D**,**E**). At the end of treatment, target and effector cells were mixed at effector/target (E/T) ratio of 3:1 in complete medium and incubated for an additional 12 h in overload conditions of monoclonal antibody (10 μg/ml). The percentage of double-positive cells present in the upper right quadrant (quadrant Q2) of the dot plots represents the percentage of RAW264.7 cells phagocytosing green-stained tumor cells. Data were obtained in triplicate and are representative of one of three experiments with similar results.

Because HER signaling in tumors regulates expression of MICA and MICB, key ligands that promote NK cell-mediated recognition and cytolysis [30], and because EGFR inhibitors enhance susceptibility to NK cell-mediated lysis by modulating expression of the NKG2D ligand ULBP-1 [30-32], we tested whether Cetuximab treatment of IGROV-1 cells modulates expression of molecules involved in NK-mediated lysis (MICA, MICB, ULBP1, ULBP2, ULBP4, CD112, CD155, ICAM-1 and HLA-E). FACS analysis of tumor cells pretreated with Cetuximab (5 μg/ml) for 72 h revealed no significant effect on expression of any these molecules (data not shown). Moreover, ^51^Cr-release ADCC assay using Cetuximab-pretreated or untreated IGROV-1 cell targets and PBMC from 12 healthy donors as effectors revealed no increase in death percentage in the pretreated tumor cells (data not shown). By contrast, in *in vitro* phagocytosis experiments carried out in overload Cetuximab antibody conditions, IGROV-1 cells pretreated with Cetuximab were more robustly phagocytosed by RAW264-7 macrophages (Figure 3) as compared to control cells. These findings raise the possibility that Cetuximab also increases susceptibility to phagocytosis of tumor cells *in vivo* and suggest that the strong anti-tumor activity observed in the CpG-ODN/Cetuximab treatment might be due in part to increased susceptibility to phagocytosis of tumor cells induced by Cetuximab.

**Figure 4:**
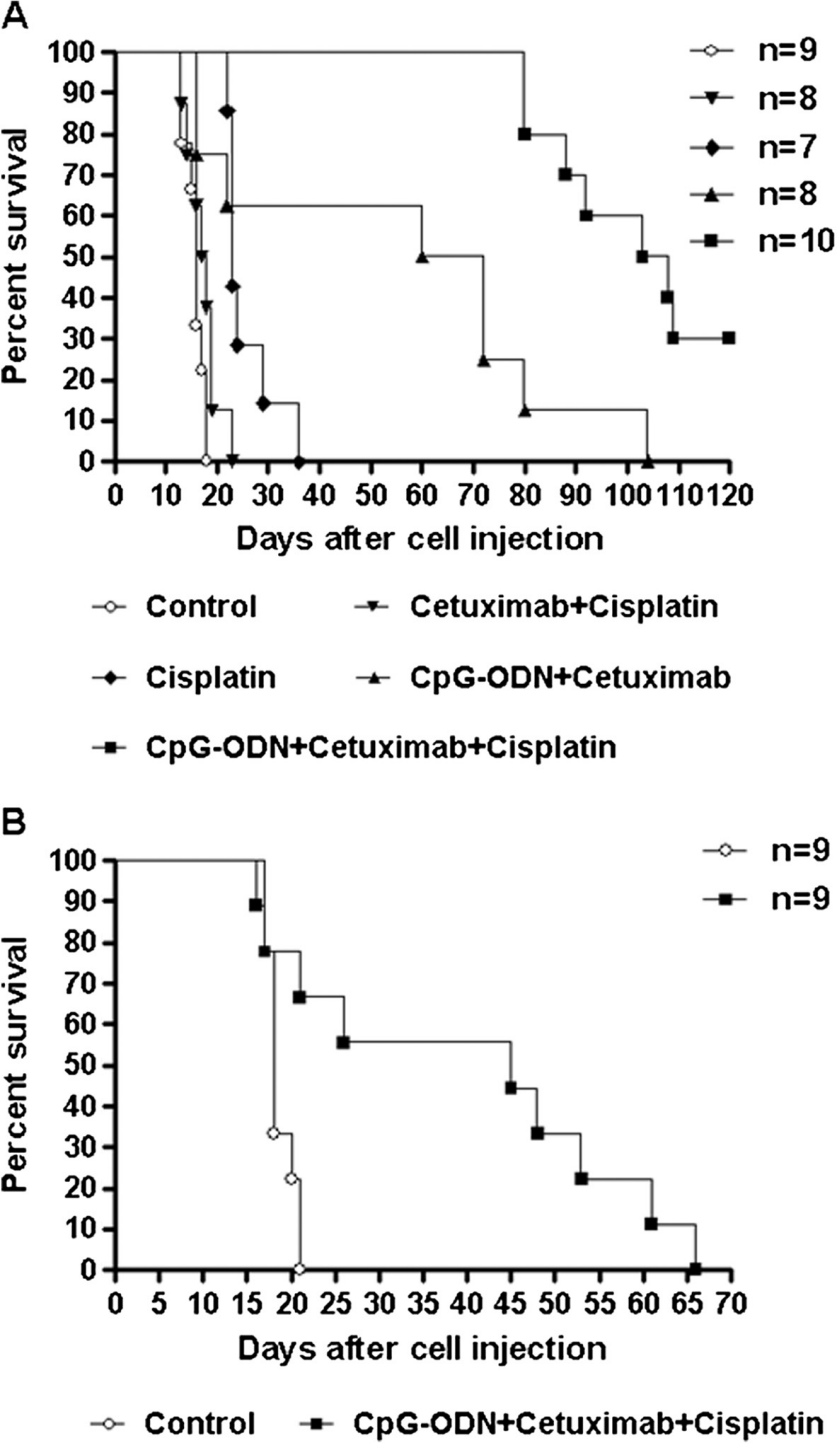
**Kaplan-Meier plot of percent survival over time in advanced-stage IGROV-1 ovarian tumor-bearing mice. **(**A**) Mice selected for the presence of evident and established ascites from a large group of mice injected i.p. 11 days before with IGROV-1 cells (mean body weight ± SEM 27.89 ± 0.84 g vs 23.00 ± 1.08 g before tumor cell injection) were treated with saline, Cisplatin (3 mg/kg, once per week), CpG-ODN (20 μg/mouse, 5 days/week for 4 weeks) plus Cetuximab (1 mg/mouse at 3- to 4-day intervals), CpG-ODN plus Cisplatin, Cetuximab plus Cisplatin, and CpG-ODN plus Cetuximab and Cisplatin. (**B**) Mice selected for more advanced-stage disease (mean body weight ± SEM 31.4 ± 0.9 g vs 24.89 ± 0.68 g before tumor cell injection) were treated with saline or CpG-ODN plus Cetuximab and Cisplatin. N = number mice/group.

EGFR inhibitors reportedly also interact with Cisplatin [33-36], although their effect on sensitivity to this drug remains undefined. Based on our recent report of synergy between CpG-ODN and Cisplatin [14], we investigated the therapeutic effect of the combination of CpG-ODN, Cetuximab and Cisplatin in mice selected for evident and established ascites from a large group of mice injected i.p. 11 days before with IGROV-1 cells (mean body weight ± SEM 27.9 ± 0.84 g vs 23.00 ± 1.08 g before tumor cell injection; increased body weight = 4.9 g). Mice were randomly divided into different groups and treated with saline, Cisplatin, CpG-ODN plus Cetuximab, CpG-ODN plus Cisplatin, Cetuximab plus Cisplatin, and CpG-ODN plus Cetuximab and Cisplatin. Saline-, Cisplatin-, or Cetuximab/Cisplatin-treated mice were euthanized on days 13 to 36 after tumor cell injection (MST 16, 23 and 18.5 days, respectively), CpG-ODN/Cetuximab-treated mice were euthanized between days 16–104 (MST 66 days; T/C% = 412.5), while 7 mice treated with the triple combination were euthanized on days 80–109, with 3 still alive at the end of experiment. Thus, survival was significantly increased (MST 105.5; T/C% 659.37; P = 0.001) compared with CpG-ODN/Cetuximab-treated mice (Figure 4A). Similar analysis in mice bearing even more advanced-stage ascites (mean body weight ± SEM 31.4 ± 0.9 g vs 24.89 ± 0.68 g before tumor cell injection; increased body weight = 6.51 g) showed that the CpG-ODN/Cetuximab/Cisplatin combination still increased survival (MST 45; T/C% 250, P = 0.0089 vs controls) (Figure 4B). Note that 6 days after the start of treatment, 6 of 9 saline-treated mice became moribund and were sacrificed.

## Conclusions

Preclinical studies in which treatment is initiated only after ascites is evident are rare and generally show a small effect on survival. Our results indicate that combination therapies to concomitantly enhance the immune response in the tumor microenvironment and target tumor cells can be effective even in advanced malignancies. Although differences in the distribution of TLR9 receptors in mice and humans as well as the enrichment of innate immune cells in athymic mice must be considered, our findings point to a promising clinical strategy for treating ovarian cancer patients with bulky ascites. Thus, clinical trials of i.p. CpG-ODN treatment in association with Cetuximab and Cisplatin might now be contemplated in ovarian carcinoma patients with bulky disease.

## Abbreviations

CpG-ODN: CpG-oligodeoxynucleotides; Poly(I):Poly(C): Polyinosinic:Polycytidylic acid; MST: Median Survival Time; TLR: Toll-like receptor; MAb: Monoclonal Antibody; EGFR: Epidermal Growth Factor Receptor; i.p.: intraperitoneal; T/C%: Treated/Control*100; ADCC: Antibody-Dependent Cellular Cytotoxicity; ADCP: Antibody-Dependent Cell-mediated Phagocytosis.

## Competing interests

The authors declare that they have no competing interest.

## Authors’ contributions

AB, MS conceived the study. MS, MDC performed the in vivo experiments. AM, AC, MS carried out the ADCC, phagocytosis assay, and the other in vitro experiments. AB, MS, AM analyzed the data. AB, drafted the manuscript. MS, AM helped to draft the manuscript. ET, NZ revised critically manuscript for important intellectual content. All the authors read and approved the final manuscript.
